# Tolterodine extended release in the treatment of male oab/storage luts: a systematic review

**DOI:** 10.1186/1471-2490-14-84

**Published:** 2014-10-27

**Authors:** Mauro Gacci, Giacomo Novara, Cosimo De Nunzio, Andrea Tubaro, Riccardo Schiavina, Eugenio Brunocilla, Arcangelo Sebastianelli, Matteo Salvi, Matthias Oelke, Stavros Gravas, Marco Carini, Sergio Serni

**Affiliations:** Department of Urology, University of Florence, Careggi Hospital, Viale S. Luca – 50134, Florence, Italy; Department of Surgical, Oncological and Gastroenterological sciences, Urology clinic, University of Padua, Padua, Italy; Department of Urology, Sant’Andrea Hospital, University ‘La Sapienza’, Rome, Italy; Department of Urology, University of Bologna, Bologna, Italy; Department of Urology, Hannover Medical School, Hannover, Germany; Department of Urology, University Hospital of Larissa, Larissa, Greece

**Keywords:** Lower urinary tract symptoms, Overactive bladder, Storage LUTS, Tolterodine, Urge incontinence, Frequency, Nocturia

## Abstract

**Background:**

Overactive bladder (OAB)/ storage lower urinary tract symptoms (LUTS) have a high prevalence affecting up to 90% of men over 80 years. The role of sufficient therapies appears crucial. In the present review, we analyzed the mechanism of action of tolterodine extended-release (ER) with the aim to clarify its efficacy and safety profile, as compared to other active treatments of OAB/storage LUTS.

**Methods:**

A wide Medline search was performed including the combination of following words: “LUTS”, “BPH”, “OAB”, “antimuscarinic”, “tolterodine”, “tolterodine ER”. IPSS, IPSS storage sub-score and IPSS QoL (International Prostate Symptom Score) were the validated efficacy outcomes. In addition, the numbers of urgency episodes/24 h, urgency incontinence episodes/24 h, incontinence episodes/24 h and pad use were considered. We also evaluated the most common adverse events (AEs) reported for tolterodine ER.

**Results:**

Of 128 retrieved articles, 109 were excluded. The efficacy and tolerability of tolterodine ER Vs. tolterodine IR have been evaluated in a multicenter, double-blind, randomized placebo controlled study in 1529 patients with OAB. A 71% mean reduction in urgency incontinence episodes was found in the tolterodine ER group compared to a 60% reduction in the tolterodine IR (p < 0.05). Few studies evaluated the clinical efficacy of α-blocker/tolterodine combination therapy. In patients with large prostates (prostate volume >29 cc) only the combination therapy significantly reduced 24-h voiding frequency (2.8 vs. 1.7 with tamsulosin, 1.4 with tolterodine, or 1.6 with placebo). A recent meta-analysis evaluating tolterodine in comparison with other antimuscarinic drugs demonstrated that tolterodine ER was significantly more effective than placebo in reducing micturition/24 h, urinary leakage episodes/24 h, urgency episodes/24 h, and urgency incontinence episodes/24 h. With regard to adverse events, tolterodine ER was associated with a good adverse event profile resulting in the third most favorable antimuscarinic. Antimuscarinic drugs are the mainstay of pharmacological therapy for OAB / storage LUTS; several studies have demonstrated that tolterodine ER is an effective and well tolerated formulation of this class of treatment.

**Conclusion:**

Tolterodine ER resulted effective in reducing frequency urgency and nocturia and urinary leakage in male patients with OAB/storage LUTS. Dry mouth and constipation are the most frequently reported adverse events.

## Background

Lower Urinary Tract Symptoms (LUTS) have a high prevalence affecting up to 90% of men over 80 years [1]. The term LUTS comprises a large group of symptoms usually divided into storage LUTS (daytime urinary frequency, nocturia, urgency, urinary incontinence), voiding LUTS (slow stream, splitting or spraying, intermittency, hesitancy, straining, terminal dribble), and post micturition LUTS (sensation of incomplete emptying, post-micturition dribble) [2].

In men, LUTS may be associated with benign prostatic obstruction (BPO) typically resulting from benign prostatic hyperplasia (BPH) or benign prostatic enlargement (BPE) [3]. Approximately half of men with histological BPH develop BPE but only 25–50% of these men have LUTS [4, 5].

OAB and storage LUTS are defined as the presence of urinary urgency, usually accompanied by frequency and nocturia, with or without urinary incontinence, in absence of urinary tract infection or other urethro-vesical dysfunctions [2]. Storage LUTS are generally a chronic condition, with a prevalence ranging from 10% to 26% [6, 7]. Male storage symptoms could be caused by bladder dysfunction (like detrusor overactivity or detrusor impaired contractility), BPO (often caused by BPE) or by a combination of both bladder dysfunction and BPO [8, 9]. In that scenario, the role of targeted therapies appears crucial. In order to obtain clinical relief of storage LUTS, an extensive counseling of patients is mandatory to evaluate all the possible treatments and their expected results since the lack of efficacy and the presence of bothering adverse events (AEs) can reduce compliance.

Behavioral therapies should be offered as first line treatment for all patients with storage LUTS. Their goal is to relieve bladder symptoms by changing voiding habits (bladder training, delayed voiding) or by improving control of urge suppression and urethral occlusion (PFMT). Nevertheless the gold standards of pharmacological therapy are antimuscarinic agents such as oxybutynin, tolterodine, fesoterodine, darifenacin, solifenacin, or trospium [10].

Antimuscarinics (m-cholinoceptor antagonists) especially block specific receptors at the level of the bladder (M_2_ and M_3_ receptors on smooth muscle cells of the detrusor) in a more or less selective manner, thereby reducing involuntary bladder contractions or altering contraction thresholds. Antimuscarinics act mainly during the urinary storage phase and decrease the activity of afferent bladder nerves [11] resulting in decreased urgency and increased bladder capacity. However, muscarinic receptors are also found in other parts of the body, including the brain, heart, gut, salivary glands, and tear ducts. First marketed antimuscarinics were limited by adverse effects, resulting in poor patient compliance and discontinuation of treatment [12].

Oxybutynin was the first antimuscarinic agent, used since the mid-70s, for the treatment of overactive bladder (OAB)/bladder storage symptoms [13]. Oxybutynin immediate release (IR) has proven efficacy for the condition [14]. However, it has a significant incidence of peripheral anti-muscarinic adverse events such as dry mouth, constipation, tachycardia, paralysis of accommodation and central nervous system side effects (cognitive dysfunction or delirium), resulting in poor compliance and early discontinuation of therapy in a large number of patients [12, 13].

More than fifteen years ago, tolterodine was developed with the aim of obtaining a better efficacy/adverse event profile and improving the compliance of patients compared to other antimuscarinic drugs. It is lesser lipid (soluble) than oxybutynin and crosses the blood–brain barrier to a lesser extent. Tolterodine is non-selective with respect to the muscarinic receptor sub-types but, as shown by data obtained from animals and healthy volunteers in the first clinical trials, showed a greater, more rapid and longer lasting effect on the bladder than on salivary glands *in vivo* [15–17].

Patient tolerability represents a fundamental parameter for the administration of antimuscarinic agents. Given the established role of frequency-dose and patient compliance and its potential effect on tolerability and efficacy, an extended release (ER) formulation was developed for several antimuscarinics. In a large systematic review and meta-analysis [18], all the comparisons among IR (drug intake 2–3 times/day) and ER formulations (drug intake once/day) showed advantages for the latter, either in terms of efficacy or safety.

Few studies investigated the effects of antimuscarinic drugs on male patients with bladder outlet obstruction and OAB/bladder storage symptoms and the results of the use of antimuscarinic agents as monotherapy were conflicting. Starting in 1994, the approach of combination therapy with α-blockers and antimuscarinics has become increasingly popular [19]. Earliest report of Athanasopoulos et al. [20] on the effects of tolterodine 2 mg twice daily combined with tamsulosin 0.4 mg once daily compared with tamsulosin alone in 25 patients showed a better QoL only in the combination therapy group with no acute urinary retention. As a result, there has been a growing interest on the use of antimuscarinics in male LUTS/BPH.

Antimuscarinics have been increasingly used in clinical practice - with caution and regular re-evaluation - in particular for selected patients with moderate to severe LUTS who have predominant bladder storage symptoms and do not have elevated post-void residual urine volumes [21, 22]. In the present review we analyzed in detail the mechanism of action of tolterodine ER and its overall safety and efficacy in the treatment of male bladder storage LUTS.

## Methods

A wide Medline search was performed including the combination of following search terms: “LUTS”, “BPH”, “OAB”, “antimuscarinic”, “tolterodine”, “tolterodine ER”. No temporary limits were adopted. IPSS, IPSS storage sub-score and IPSS QoL (International Prostate Symptom Score) were the validated efficacy outcomes. In addition, the numbers of urgency episodes/24 h, urgency incontinence episodes/24 h, incontinence episodes/24 h and pad use were considered. We also evaluated the most common adverse events (AEs) reported for tolterodine ER in selected studies.

## Results

**Figure 1:**
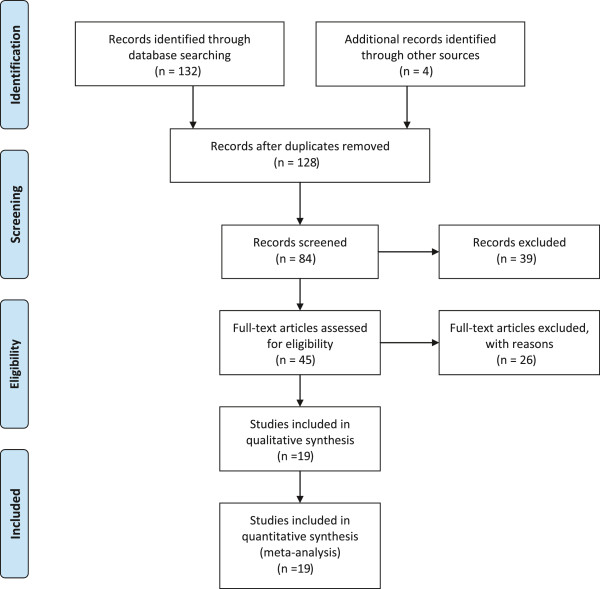
**Flowchart of literature searches according to PRISMA statement.**

Out of 128 retrieved articles, 109 were excluded for missing or incomplete data, deficiency in methodology (several biases not included), assessment of clinical outcomes without validated instruments. the total flowchart of literature searches is summarized in Figure 1.

## Mechanism of action of tolterodine

### Muscarinic receptors

Five sub-types of muscarinic receptors are presented in the human tissues: even if all these receptors can be found in several tissues, including epithelial cells of the bladder and the salivary glands and nerve cells of the central or peripheral nervous systems, the M_2_ and M_3_ are predominantly expressed in detrusor smooth muscle cells [23]. Detrusor contractions are stimulated by the activity of acetylcholine on muscarinic receptors on smooth muscles cells of the bladder.

Tolterodine is a competitive muscarinic receptor antagonist with relative functional selectivity for bladder muscarinic receptors. It is metabolized in microsomes of the human liver by cytochromes P450 (CYP2D6 and CYP3A4) to two primary metabolites: 5-hydroxymethyl tolterodine (5-HMT) (labcode DD 01; PNU-200577) and *N*-dealkylated tolterodine [23, 24]. With the exception of 5-HMT, metabolites of tolterodine are not considered to contribute to the therapeutic effect.

*In vitro* studies in guinea-pig detrusor strips [25] showed a simple competitive blockade of the bladder muscarinic receptors in a concentration-dependent manner after carbachol-induced contractions. Tolterodine was equipotent to oxybutynin and acted as an effective and competitive muscarinic receptor antagonist also in human isolated urinary bladder. Radioligand binding studies in tissue homogenates showed that the affinities determined and expressed as the dissociation constants (k_i_), for tolterodine in human bladder were comparable to those in the guinea-pig bladder [25].

### Selectivity profile

The binding affinities determined in the bladder were similar to those in the heart, which can be assumed to contain only muscarinic M_2_ receptors [25].

Tolterodine and 5-HMT show functional selectivity for the bladder over the salivary glands *in vivo*. In the anaesthetized cats, intravenous injection of tolterodine and 5-HMT resulted in dose-dependent inhibition of acetylcholine-induced urinary bladder contractions and electrically induced salivation. The effect on urinary bladder contractions occurred at significantly lower doses than the effect on salivary secretion, showing favorable tissue selectivity [25–27]. In 2008 Olshansky et al. showed an increase in mean heart rate per 24 hours of ≥5 beats per minute higher with tolterodine than with placebo (p = 0.0114) [28]. Neither oxybutynin nor tolterodine showed clinically significant effects on the heart rate [25, 27].

The selectivity profiles in vivo were reflected in the radioligand binding studies. Thus, the affinity profile of tolterodine (cerebral cortex ≥ heart ≈ urinary bladder > parotid gland) differed from those of oxybutynin (cerebral cortex ≈ parotid gland > heart ≈ urinary bladder).

### Pharmacokinetics

The pharmacokinetic properties of tolterodine are influenced by the CYP2D6 polymorphism. The lack or strongly reduced activity of this liver enzyme characterizes poor metabolizers. In these individuals, the active metabolite 5-HMT cannot be formed and the pharmacological effects are mediated exclusively by tolterodine. As shown in a clinical study by Brynne et al. [29], tolterodine was rapidly absorbed in both extensive and poor metabolizer, and the pharmacodynamic effects of tolterodine were not generally influenced by metabolic phenotype. Thus, the same dosage can be used irrespective of CYP2D6 phenotype.

Pharmacokinetic equivalence was demonstrated between IR tolterodine tablets 2 mg twice daily and ER capsule formulation of tolterodine 4 mg once daily (AUC_24_). In addition, tolterodine ER resulted in less serum drug level fluctuation and sustained drug release over 24 hours [27]. This translates into more constant serum concentrations and, in theory, also into better tolerability for patients.

The clearance of tolterodine was considerably lower in patients with liver cirrhosis or impaired renal function [creatinine clearance 10–30 ml/min (0.6 to 1.8 L/h)] compared to healthy volunteers [30].

## Discussion

### Safety and efficacy of tolterodine Er

#### Tolterodine ER vs. Tolterodine IR

Tolterodine intermediate release (IR) was firstly developed and tested in several randomized, double-blind, placebo controlled study which led to drug approval by the FDA in 1998. The efficacy and tolerability of tolterodine IR and oxybutynin IR were found to be comparable [14]. Dry mouth was the only adverse event that occurred significantly more often in patients treated with tolterodine IR (1 mg bid, 30%; 2 mg bid, 48%) in comparison to patients of the placebo group; however, only 3% of the tolterodine IR treated subjects withdrew from treatment because of dry mouth [31].

Regardless of the positive results of the registration trials and confirmation of the excellent efficacy in randomised phase IV studies [32–34], Pfizer laboratories developed an extended release (ER) formulation (approved by the FDA in 2000) to improve patient compliance and to decrease the dry mouth rate which was thought to be dependent from the peak plasma levels of the drug. Tolterodine ER uses a drug delivery system that contains soluble microspheres. The drug is slowly released as the outer layer of the microsphere dissolves, leading to consistent delivery of drug over a 24-hour period [35].

The efficacy and tolerability of tolterodine ER have been evaluated in a multicenter, double-blind, randomized placebo controlled study in 1529 patients with OAB [13, 36]. A significant clinical advantage in terms of clinical efficacy and tolerability was associated with tolterodine ER treatment. A 71% mean reduction in urgency incontinence episodes was found in the tolterodine ER group compared to a 60% reduction in the tolterodine IR group (p < 0.05). The incidence of dry mouth was 23% for tolterodine ER versus 30% for tolterodine IR. The overall rate of dry mouth was 23% lower with tolterodine ER than with tolterodine IR. The incidence of other adverse events such as dizziness (ER 2% vs. IR 2%), constipation (ER 7% vs. IR 6%) and somnolence (ER 3% vs. IR 3%) were similar between the treatment groups and comparable with placebo. This pivotal study suggested an improved clinical advantage of tolterodine ER over the IR formulation of the drug in terms of efficacy and tolerability.

### Tolterodine in combination therapies for OAB/LUTS

The theoretical concern about a negative effect on post-void residual urine or even urinary retention has influenced the use of antimuscarinics for the management of storage LUTS in male patients independent of studies showing no increased risk of urinary tract retention in patients with benign prostatic obstruction. The combined use of antimuscarinics and other drugs currently available for LUTS, including α-blockers, 5α-reductase inhibitors or botulinum toxin A in addition to the introduction of ß_3_-agonists has recently been investigated to overcome these limitations [37–40].

Few studies have evaluated the clinical efficacy of α-blocker/tolterodine combination therapy [37–39]. The majority are add-on studies, in which tolterodine has been added to an existing α_1_-blocker therapy. The “Tolterodine and Tamsulosin in Men With LUTS Including OAB: evaluation of Efficacy and Safety” (TIMES) study showed that patients treated with tolterodine/tamsulosin combination therapy, but not with tamsulosin, tolterodine or placebo alone, had a significant treatment benefit as defined by the patient perception questionnaire (80% vs. 71%, 65%, and 62%, respectively) [37, 39]. At the end of the study period (12 weeks), only combination therapy significantly improved total IPSS and QoL as well as the IPSS storage sub-score. The TIMES study also identified a subgroup of patients with a PSA value <1.3 ng/ml or prostate volume <29 ml who also significantly profited from tolterodine monotherapy with regard to storage symptom reduction [37, 39]. In patients with large prostates (prostate volume >29 cc) only the combination therapy significantly reduced 24-h voiding frequency (2.8 vs. 1.7 with tamsulosin alone, 1.4 with tolterodine alone, or 1.6 with placebo). Adverse events of antimuscarinics (e.g. dry mouth or constipation) occurred in the combination therapy group more often than in patients receiving α_1_-blocker monotherapy. There was no significant or clinically relevant increase in post-void residual urine or acute urinary retention when the combination treatment arm was compared with mono-therapy of the individual drugs [39].

Another investigational combination therapy with tolterodine ER was with 5α-reductase inhibitors. In particular, Chung et al. demonstrated that in men with persistent OAB symptoms after at least 6 months of treatment with dutasteride the addition of tolterodine ER allowed to significantly reduce frequency and urgency, such as severe OAB episodes and night time voiding (nocturia). Storage LUTS (IPSS storage sub-score) were remarkably reduced from 9.8 to 4.5 (p < 0.001). Regarding tolerability, 7.5% of men experienced dry mouth, but no patient developed urinary retention [40].

The efficacy and safety of tolterodine in combination therapies was reviewed by Athanasopoulos et al. in 2011, concluding that combination therapy was effective and the risk of urinary retention was minimal [41].

Mirabegron, a novel ß_3_-adrenoceptor agonist, has recently been approved for the treatment of OAB symptoms and is the first of a new class of compounds with a mechanism of action that is different from antimuscarinic agents. Mirabegron represents a new option for the management of OAB, has a comparable efficacy and a better tolerability when compared to tolterodine 4 mg ER in a large clinical trial dataset in OAB/storage LUTS patients. However, further studies should assess its long term safety and efficacy and the possible role in specific group of patients as male patients with LUTS and benign prostatic obstruction, either alone or in combination with antimuscarinics (e.g. tolterodine ER) [42, 43].

### Tolterodine ER vs. other antimuscarinics

Since its introduction in clinical practice, tolterodine has been the active comparator in several studies. The first comparator trial using tolterodine ER was the “Overactive Bladder: Performance of Extended Release Agents” (OPERA) study [44] which compared the efficacy and tolerability of tolterodine ER (4 mg daily) and oxybutynin (10 mg daily). No significant difference was observed in the number of urgency incontinence episodes (tolterodine 20.9% vs. oxybutynin 26.7%) or the total dry rate (tolterodine 16.8% vs. oxybutynin 23%).

Regarding adverse events, the most common side effect in each group was dry mouth, with 29.7% of the patients receiving oxybutynin vs. 22.3% of those receiving tolterodine (p = 0.02). Other adverse events were similar in magnitude and frequency in both groups [18, 35]. A recently published study tested the efficacy and tolerability of tolterodine ER versus solifenacin [45]. The STAR-study compared flexible dosing of solifenacin versus tolterodine 4 mg in the primary outcome criteria (change in number of micturictions per 24 hours). Solifenacin flexible dosing proved to be superior to tolterodine ER in reducing the numbers of urgency episodes/24 h (-2.85 vs. -2.42), urgency incontinence episodes/24 h (-1.42 vs. -0-83), incontinence episodes/24 h (-1.60 vs. 1–11), and pad use (-1.72 vs. -1.19). Dry mouth and constipation were significant more common in the solifenacin arm (18.2 vs. 14.5% and 3.0 vs. 1.2%, respectively), although they were mainly of mild to moderate severity [18, 45].

In 2008 Chapple et al. compared the antimuscarinic fesoterodine 4 mg or fesoterodine 8 mg once daily to placebo in a randomized controlled trial and included an active control arm tolterodine ER 4 mg [46]. Fesoterodine 8 mg outperformed tolterodine 4 mg with regard to the median change from baseline in number of UUI episodes (p < 0.05) and volume voided per micturition (p < 0.05), while similar efficacy was shown for fesoterodine 4 mg and tolterodine 4 mg. Fesoterodine 4 mg and tolterodine ER 4 mg had a similar safety profile, while fesoterodine 8 mg was associated with significantly higher rates of dry mouth (p < 0.0001) and dry eyes (p = 0.02) compared to tolterodine 4 mg [18]. In 2013 Ginsberg et al. [47] compared the efficacy of fesoterodine 8 mg vs tolterodine 4 mg ER for OAB symptoms in terms of patient-reported outcomes in both men and women, supporting the superiority of fesoterodine 8 mg over tolterodine 4 mg ER in improving severe urgency and symptom bother in men.

The EAU Guidelines on Urinary Incontinence recently evaluated and reported data from the Agency for Healthcare Research and Quality (AHRQ) review which included a specific section addressing comparisons of antimuscarinic drugs [48, 49] (Table 1). They concluded that there was no evidence that any one antimuscarinic, including tolterodine ER, improved quality of life more than another agent and there is no consistent evidence for the superiority of one antimuscarinic agent over another for the overall efficacy or discontinuation rate. However, the recently published studies comparing tolterodine with fesoterodine have not been included in this analysis.

**Table 1:** **Comparison of tolterodine ER versus other antimuscarinics as reviewed in the 2012 AHRQ review** [49]

Experimental drug versus standard drug	No. of studies	Patients	Relative risk (95% CI)
**Efficacy (cure of UI)**			
Fesoterodine vs tolterodine ER	2	3312	1.1 (1.04-1.16)
Oxybutynin ER vs tolterodine ER	3	947	1.11 (0.94-1.16)
Solifenacin vs tolterodine ER	1	1177	1.2 (1.08-1.34)
**Discontinuation due to adverse events**			
Solifenacin vs tolterodine ER	3	2755	1.28 (0.86-1.91)
Fesoterodine vs tolterodine ER	4	4440	1.54 (1.21-1.97)

Efficacy was defined as the achievement of urinary continence.

**Figure 2:**
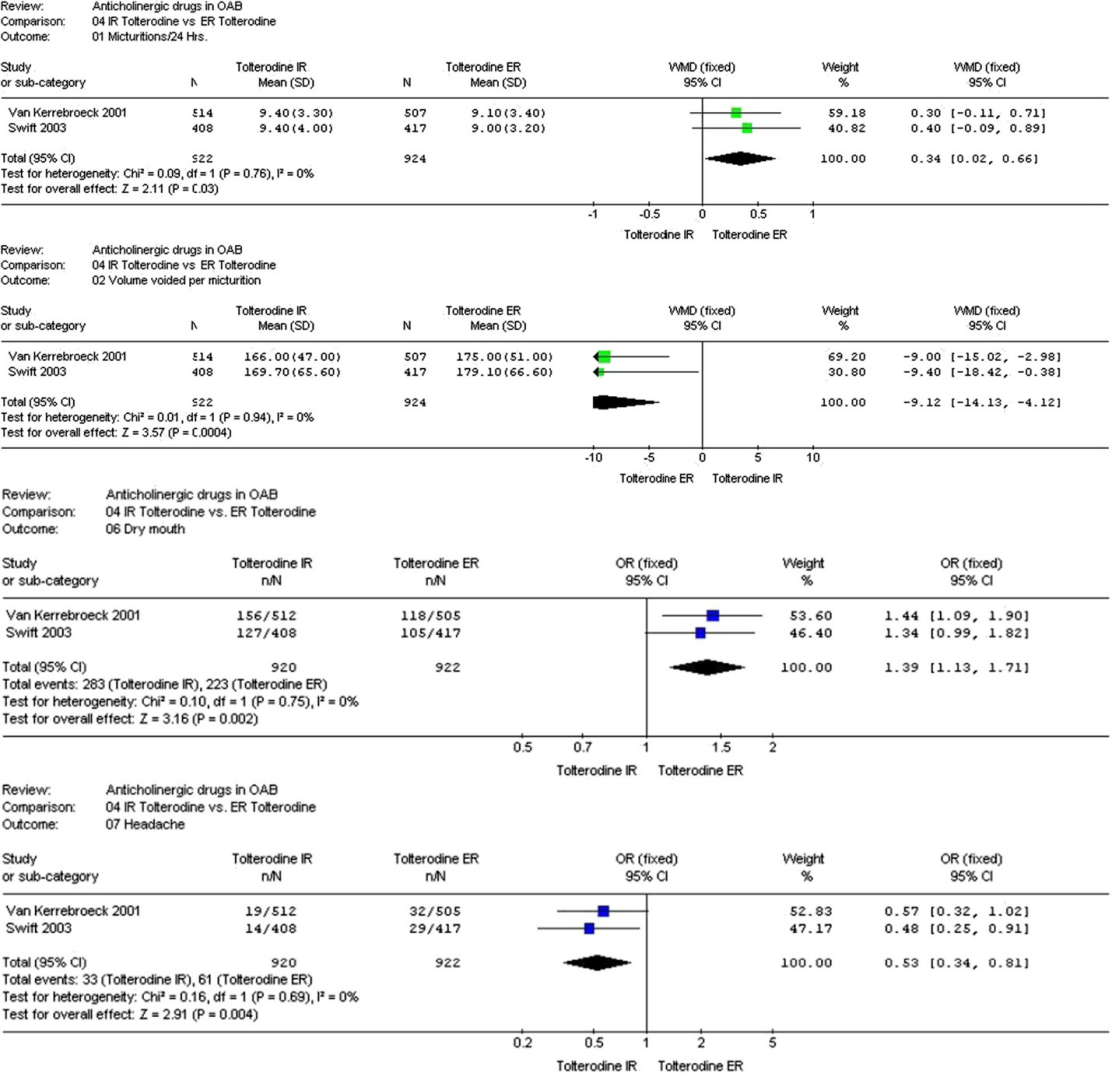
**Forest plots of efficacy and safety after IR and ER tolterodine.** 3a: micturitions/24 Hrs; 3b: volume voided per micturition; 3c: dry mouth; 3d: headache. (License number 3340911442671 of Mar 2, 2014 between Elsevier and Dr. G. Novara).

A recent network meta-analysis evaluating tolterodine in comparison with other antimuscarinic drugs demonstrated that tolterodine ER was significantly more effective than placebo in reducing micturition/24 h (-0.76; *p* <0.001), urinary leakage episodes/24 h (-0.36; *p* <0.001), urgency episodes/24 h (-0.77; *p* <0.001), and urgency incontinence episodes/24 h (-0.34; *p* <0.001) [48]. With regard to adverse events, the same article demonstrated that tolterodine ER was associated with a good adverse event profile resulting in the third most favorable antimuscarinic out of 21 analyzed antimuscarinic drugs, following oxybutynin topical gel 100 mg/g per day and solifenacin 5 mg per day [50] (Figure 2).

## Conclusions

Tolterodine is an effective muscarinic receptor antagonist, with a receptor affinity comparable to oxybutynin in the bladder, and a remarkably lower affinity than oxybutynin in the parotid gland. Immediate-release tablets 2 mg twice daily and extended-release tablets 4 mg once daily have a comparable pharmacokinetic profile.

Tolterodine ER resulted effective in reducing frequency urgency and nocturia and urinary leakage in patients with OAB/storage LUTS. Dry mouth and constipation are the most frequently reported adverse events. The good safety profile, which allow to minimize treatment withdrew, and the adequate effectiveness in the management of storage LUTS, are the strengths of Tolterodine ER.

Further RTCs are needed to identify the best candidates for the treatment with tolterodine ER and to tailor promising combination therapies with other drugs currently available for male LUTS.
